# Layered Double Hydroxide Reshapes the Immune Microenvironment of Rheumatoid Arthritis through Small Mothers against Decapentaplegic 5

**DOI:** 10.34133/bmr.0176

**Published:** 2025-03-28

**Authors:** Dengju Li, Yawei Sun, Guangxian Liu, Changxing Liu, Guojiang Zhang, Haojue Wang, Shui Sun, Senbo An

**Affiliations:** ^1^ Department of Joint Surgery, Shandong Provincial Hospital Affiliated to Shandong First Medical University, Jinan, Shandong 250021, China.; ^2^ Shandong Key Laboratory of Reproductive Medicine, Department of Obstetrics and Gynecology, Shandong Provincial Hospital Affiliated to Shandong First Medical University, Jinan, Shandong 250021, China.; ^3^ Department of Orthopaedic, Shandong Provincial Hospital Affiliated to Shandong First Medical University, Jinan, Shandong 250021, China.; ^4^ Department of Joint Surgery, Shandong Provincial Hospital, Shandong University, Jinan, Shandong 250012, China.; ^5^ Orthopaedic Research Laboratory, Medical Science and Technology Innovation Center, Shandong First Medical University & Shandong Academy of Medical Sciences, Jinan, Shandong 250117, China.

## Abstract

Persistent synovitis is a pivotal pathological feature of rheumatoid arthritis (RA). However, the current rheumatoid drugs are accompanied by severe side effects and have limited anti-inflammatory capabilities. In this work, we designed a bioactive material—folic acid modified layered double hydroxides (FA-LDH), aiming at targeting M1 macrophages and modulating macrophage repolarization. The in vitro experiment showed that FA-LDH mitigated the release of proinflammatory cytokines and promoted the expression of M2 macrophage markers. In terms of the action mechanism, FA-LDH modulated the nucleocytoplasmic transport of the small mothers against decapentaplegic 5 (Smad5) protein by adjusting the pH within the immune microenvironment. Subsequently, relying on the interaction between phospho-Smad5 (pSmad5) and p65, the nuclear factor kappa B signaling pathway was down-regulated through inhibiting nuclear transport of p65. Additionally, FA-LDH exhibited excellent targeting capability toward M1 macrophages and strong accumulation capacity in inflamed joints. In vivo experiment showed that FA-LDH could relieve swelling of limbs, reduce the infiltration of inflammatory cells, and protect joint cartilage and subchondral bone structure in collagen-induced arthritis mice. In summary, this work introduces a strategy for utilizing bioactive FA-LDH in the treatment of RA, highlighting the potential of FA-LDH to alleviate inflammation and reshape the immune microenvironment through the pSmad5/p65 axis.

## Introduction

Rheumatoid arthritis (RA) is a chronic autoimmune disease caused by immune system disorder [1]. RA is characterized by progressive inflammation and persistent synovitis, leading to bone and cartilage destruction, ultimately resulting in function loss [2]. Therapeutic strategies encompass the use of traditional synthetic disease-modifying antirheumatic drugs, nonsteroidal anti-inflammatory drugs, and glucocorticoids, which can ameliorate symptoms and decelerate the progression of arthritis to some extent [3]. However, the necessity for high dosages and frequent administration to achieve efficacy often causes undesirable side effects [4]. Biological agents have been developed to prevent joint destruction by suppressing cytokine levels [3]. Although biologics demonstrate effective therapeutic outcomes, they are associated with high costs and serious risks, such as the reactivation of tuberculosis and severe infections [5]. Consequently, there is an urgent need for new antirheumatic drugs with higher efficacy and safety.

Macrophages play key roles in RA. Activated macrophages (M1 macrophages) produce a series of inflammation cytokines, such as tumor necrosis factor-α (TNF-α) and interleukin-1β (IL-1β) [6]. A mount of macrophages with M1 phenotype are infiltrated in synovial tissue of RA joints [7]. The release of inflammatory cytokines by M1 macrophages promotes RA progression. M2 macrophages secrete anti-inflammatory cytokines, such as IL-10 and transforming growth factor-β (TGF-β) [8]. TGF-β also participates in a variety of biological processes, such as bone repair [9]. M1 and M2 macrophage polarization dynamically adapts to changes in the microenvironment, exhibiting the plasticity of macrophage polarization [7]. Anti-inflammatory and immunosuppressive responses can be triggered by inducing the transformation of M1 macrophages into M2 macrophages [10]. As one of the traditional inflammatory routes, the nuclear factor kappa B (NF-κB) signaling pathway is essential for macrophage polarization and RA development [11]. Sustained activation of the NF-κB signaling pathway leads to an elevated expression of inflammatory cytokines, such as TNF-α and IL-1β, which can promote the polarization of macrophages toward the M1 phenotype, increase damage to the extracellular matrix and degradation of the cartilage, and exacerbate RA ultimately [7,12]. Duan and Li [13] created a hybrid nanocarrier combined with NF-κB-targeting siRNA and methotrexate. It could accumulate in RAW 264.7 cells and lower the levels of proinflammatory cytokines (IL-1β and TNF-α) from the serum of collagen-induced arthritis (CIA) mice. Insulin-like growth factor binding protein 3 can inhibit the progression in CIA mice by suppressing NF-κB signaling and inhibiting the levels of proinflammatory cytokines [14]. It has emerged as an efficient therapeutic to treat RA by inhibiting the NF-κB signaling pathway and changing the predominant M1 macrophages in arthritic joints to M2 macrophages [8,15].

Layered double hydroxides (LDH) are a kind of inorganic material with mild alkalinity, composed of hydroxide layers and metal ion interlayers, exhibiting biocompatibility, biodegradability, and pH sensitivity [16]. The LDH has been extensively investigated in the field of biomedical engineering such as tissue engineering and drug delivery [16,17]. Furthermore, the mild alkalinity of LDH nanosheets effectively suppressed the osteoclast viability and promoted bone formation in osteoporosis model mice by neutralizing excess H^+^ in the acidic microenvironment [18]. Besides, many studies have reported that alkaline biomaterials exhibit anti-inflammatory properties [19,20]. It is widely recognized that the acid-neutralizing capacity of biomaterials has potential anti-inflammatory properties. However, the precise cellular response to biomaterials with acid-neutralizing capacities remains unclear. Additionally, there is a paucity of studies examining the immunoregulatory role of LDH in macrophages.

Small mothers against decapentaplegic 5 (Smad5) acts as a pH messenger and maintains bioenergetic homeostasis by regulating the cytoplasmic metabolic machinery [21]. Basic pH dissociates protons from the charged amino acid clusters within the MH1 domain of Smad5, prompting its relocation from the nucleus to the cytoplasm. Under acidic conditions, Smad5 nuclear export is inhibited, leading to its nuclear accumulation. In brief, the nuclear translocation of Smad5 can be modulated by the pH of the cellular microenvironment. Whether nuclear translocation of Smad5 is associated with inflammatory pathways is of interest to us. p65 is an important transcription factor in the NF-κB pathway. It is reported that the interaction exists between the Smad5 and p65 [22,23]. Overexpression of phospho-Smad5 (pSmad5) in osteoclasts could activate p65, thereby promoting its nuclear translocation; in contrast, inhibition of the expression of pSmad5 could reduce p65 activation [24]. However, whether changes in pH can regulate nuclear transport of pSmad5 is unknown. Furthermore, whether LDH can inhibit the NF-κB pathway by regulating the pSmad5/p65 axis is a topic worth further investigating.

**Fig. 1.**
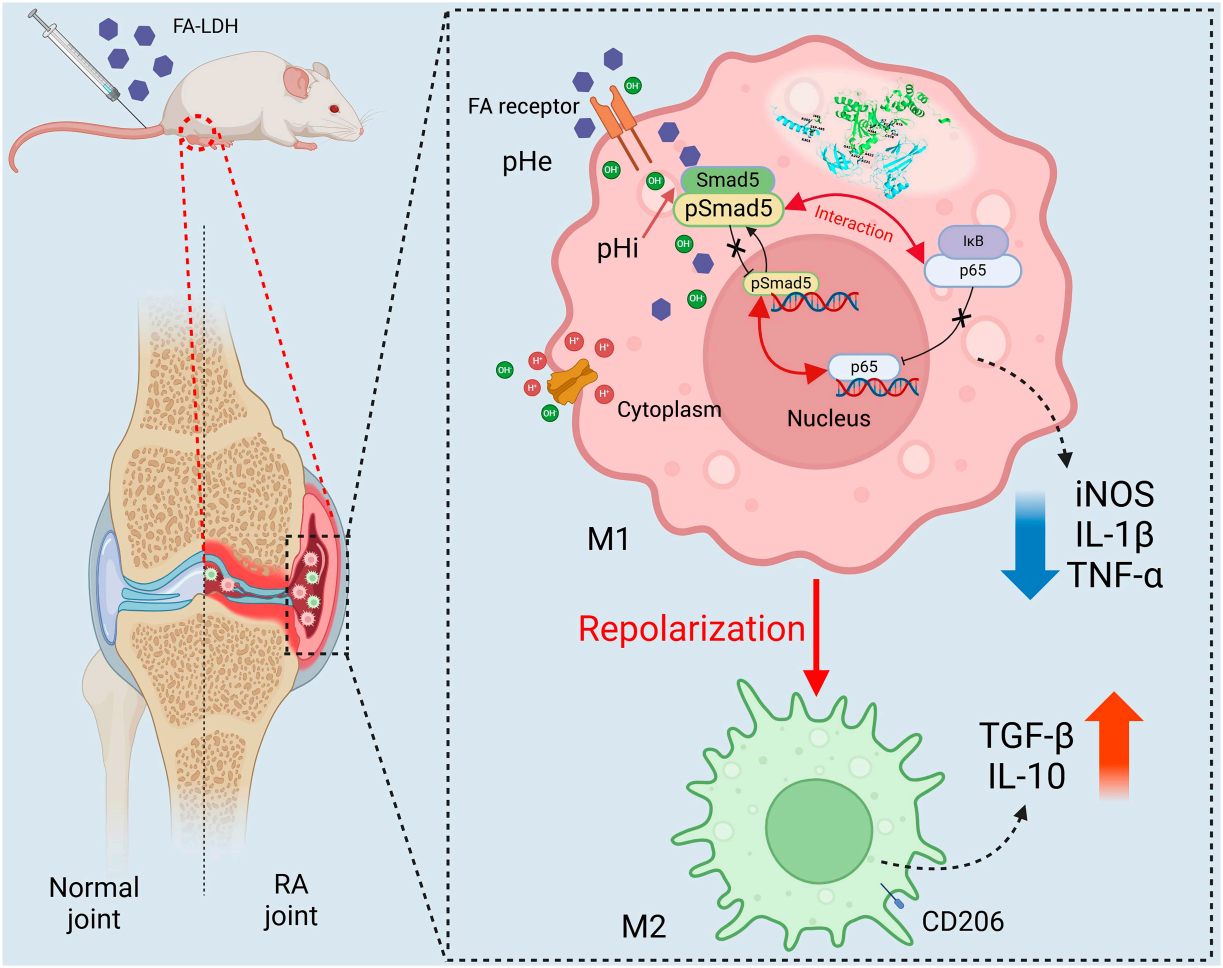
M1 targeted FA-LDH regulates the repolarization of macrophages within RA therapy.

In this study, LDH was synthesized using CaCl_2_, AlCl_3_, and NaOH and functionalized with folic acid (FA). Its therapeutic potential for RA was investigated subsequent to its physicochemical characterization. In vitro, LDH could transform macrophages from M1 to M2 phenotype in the LPS-activated macrophages model. Transcriptome sequencing was employed to elucidate the immunoregulatory characteristics of LDH. The findings revealed a substantial down-regulation of the NF-κB signaling pathway. Additionally, LDH modulated the nuclear translocation of Smad5 and p65 in macrophages by regulating the pH of the immune microenvironment, which may be the key factor of its immunoregulatory properties, as summarized in Fig. 1. The in vivo imaging system (IVIS) staining confirmed that FA modified layered double hydroxides (FA-LDH) can effectively accumulate in inflamed joints. Finally, the therapeutic effect and safety of LDH was evaluated in CIA mice. This study demonstrated the potential applicability of LDH as a therapeutic intervention for RA, providing a novel insight into the biofunction of LDH.

## Materials and Methods

### Synthesis of LDH and FA-LDH

LDH was synthesized using the hydrothermal method. Briefly, 15 ml of CaCl_2_ (0.6 M) was mixed with 15 ml of AlCl_3_ (0.2 M). Then, NaOH solutions with concentrations of 0.075, 0.15, and 0.3 M were added dropwise with mechanical stirring, respectively. The mixture was heated at 100 °C for 16 h under magnetic stirring to obtain CaAl-LDH, abbreviated as LDH. The resultant LDH solution was washed by suction filtration to remove unreacted ions and large particles. FA (1 mg) was added in 10 ml of LDH solution under continuous magnetic stirring at 60 °C for 24 h to obtain the FA-LDH solution. The resultant FA-LDH solution was washed by suction filtration to remove unreacted ions and large particles, yielding a stable FA-LDH solution for further use.

### Characterization of LDH

X-ray diffraction (XRD) patterns were procured using a diffractometer (SmartLab, Rigaku, Japan) equipped with Cu Kα radiation, characterized by a wavelength of 0.1541 nm. XRD was conducted within a scanning range of 5° to 80° employing a scanning rate of 5.0° min^−1^ and a step size of 0.02°. The morphological characterization of LDH and FA-LDH was conducted using transmission electron microscopy (TEM, 220 V, JEM- F200, JEOL, Japan). Hydrodynamic diameter was assessed through dynamic light scattering (DLS, Zetasizer Nano ZS90, Malvern, UK). Zeta potential analysis was carried out using Zetasizer Nano ZS90 (Malvern, UK). The concentrations of Ca^2+^ and Al^3+^ in LDH were quantified through inductively coupled plasma–optical emission spectrometry (ICP-OES; iCAP 7400, Thermo Fisher, USA).

### Cell cytotoxicity assay

RAW 264.7 cells were cultured in 96-well plates at a seeding density of 1 × 10^4^ cells per well and incubated at 37 °C for 24 h. Subsequently, LDH was introduced into the wells and co-incubated for 24 or 72 h. The medium was discarded and the cells were washed twice with phosphate-buffered saline (PBS). The cells were exposed to different formulations diluted using cell culture medium for 48 h. At the conclusion of the treatment phase, each well was supplemented with 100 μl of Cell Counting Kit-8 (CCK-8) solution, diluted 10-fold. After 4 h, the absorbance of CCK-8 was quantified at 450 nm using a microplate reader. This approach allowed for a detailed examination of the cellular response to the given treatments.

### Live/dead staining assay

RAW 264.7 cells were cultured in 24-well plates at a seeding density of 1 × 10^5^ cells per well and incubated at 37 °C for 24 h. The cells were incubated at room temperature for 1 h with 1 ml of a working solution of 2 mM calcein-AM and 4 mM ethidium homodimer-1. Fluorescence was visualized using excitation wavelengths of 488 nm for calcein-AM, which emits green fluorescence and labels live cells, and 568 nm for ethidium homodimer-1, which emits red fluorescence and labels dead cells.

### Immunofluorescence staining assay

RAW 264.7 macrophages were exposed to LPS stimulation for 24 h, followed by an additional 24-h treatment with various groups. Subsequently, the cells were fixed, permeabilized, blocked, and incubated with primary antibodies targeting TNF-α, CD206, Smad5, pSmad5, and p65. This was followed by incubation with the corresponding secondary antibodies. Cells positive for TNF-α or CD206 were classified as M1 or M2 macrophages, respectively.

### Western blot analysis

RAW 264.7 macrophages were cultured in 6-well plates at a seeding density of 5 × 10^5^ cells per well, using complete Dulbecco’s modified Eagle’s medium as growth medium, stimulated with LPS for 24 h, pretreated with various treatments (LDH and FA-LDH) for 24 h, and washed twice with 1× PBS. Total protein was isolated from the cultured cells utilizing a whole cell lysis buffer supplemented with a protease inhibitor cocktail (Sigma-Aldrich). The lysate was subjected to centrifugation at 12,000 rpm for 15 min to harvest protein in the supernatant. Protein concentration was ascertained using the bicinchoninic acid assay. The proteins, once solubilized in sodium dodecyl sulfate (SDS)-sample loading buffer, were separated via 10% SDS-PAGE (polyacrylamide gel electrophoresis) and transferred onto 0.22-μm polyvinylidene fluoride (PVDF) membranes. The membranes were blocked with 5% skim milk in 1 × tris-buffered saline with Tween 20 at room temperature for 1 h, followed by an overnight incubation at 4 °C with the primary antibodies (β-actin, 1:2,000; GAPDH, 1:2,000; p65, 1:1,000; p50, 1:1,000; iNOS, 1:1,000; IL-1β, 1:1,000; IL-10, 1:1,000; and TGF-β, 1:1,000). The membranes were then incubated with the secondary antibodies for 1 h at room temperature, and antibody reactivity was visualized. The quantification of gray values of the bands was performed using ImageJ software.

### Immunoprecipitation

RAW 264.7 cells were harvested and incubated with immunoprecipitation (IP) lysis buffer in an ice bath for 30 min. The lysates were centrifuged at 13,000 rpm for 10 min. The supernatants were carefully collected and incubated overnight with a primary antibody targeting pSmad5 (1:100). Subsequently, Protein A/G PLUS-Agarose (sc-2003, Santa Cruz Biotechnology) was incubated for 4 h with the mixture. The mixture was centrifuged at 3,000 rpm for 1 min to isolate the target protein, which was earmarked for Western blot analysis.

### Transcriptome sequencing and data analysis

RAW 264.7 macrophages were stimulated with LPS for 24 h, followed by treatment with LDH for 24 h. Then, RNA was isolated using RNAiso Plus (9109; Takara) and stored at −80 °C for sequencing. The Illumina HiSeq X10 platform (Illumina, USA) was used for sequencing. Gene expression values were transformed and normalized using the fragments per kilobases per million reads method. Library preparation and sequencing were conducted by Shanghai OE Biotech. Co., Ltd.

### qRT-PCR analysis

RAW 264.7 macrophages were cultured in 6-well plates at a density of 5 × 10^5^ cells per well and stimulated with LPS for 24 h. The cells were exposed to various treatments (LDH and FA-LDH) for 24 h. Total RNA was isolated from cells using RNAiso Plus (9109; Takara) according to the manufacturer’s instructions. The cDNA was amplified using SYBR Green dye (AG11701; Accurate Biology). The primer sequences are listed in Table S1. The levels of mRNA, including IL-1β, TNF-α, CD206, and IL-10, were measured using quantitative reverse transcription polymerase chain reaction (qRT-PCR) with cycling conditions of 40 cycles at 95 °C for 5 s and 60 °C for 30 s (CFX-Connect, BIO-RAD, USA).

### Molecular docking techniques

pSmad5 and p65 were modeled via AlphaFold3 using residues 1 to 320 of p65 and residues 1 to 134 and 256 to 465 of pSmad5. Global RAnge Molecular Matching (GRAMM) was used for protein–protein docking, and docking conformation was analyzed by PDBePISA (https://www.ebi.ac.uk/msd-srv/prot_int/).

### Animals

DBA/1J mice (male, 7 to 8 weeks old) were maintained in a sterile environment and allowed free access to food and water. The use of mice in all experiments was within the protocol permitted by the ethics committee of Shandong Provincial Hospital Affiliated to Shandong First Medical University, and all experiments were carried out in accordance with the requirements of the National Act on the Use of Experimental Animals (The People’s Republic of China).

### Mice model of CIA

The CIA mouse model was established using 2 emulsions. Emulsion A was formulated by combining 2 mg/ml of bovine type II collagen with an equivalent volume of Complete Freund’s Adjuvant. This mixture was then subjected to overnight stirring in an ice bath to ensure thorough mixing and emulsion formation. Emulsion B was prepared in a similar manner, but using Incomplete Freund’s Adjuvant instead. On day 0, mice were administered 0.1 ml of emulsion A via intravenous injection in the tail. On day 21, a booster injection of emulsion B was given. The progression of arthritis in the mice was monitored daily. The arthritis scores were recorded. The severity of arthritis in all 4 paws was assessed using a scale ranging from 0 to 4, as follows: 0 denotes the absence of erythema or swelling; 1 indicates the presence of erythema and mild swelling; 2 signifies erythema and mild swelling extending from the ankle to the tarsals; 3 denotes erythema and moderate swelling extending from the ankle to the metatarsal joints; and 4 indicates erythema and severe swelling encompassing the ankle, foot, and digits, or limb ankylosis. The maximum possible arthritis score for each mouse was set at 16.

### IVIS imaging

To assess the targeting efficiency of FA-LDH toward polarized macrophages within the inflamed synovium, we administered Cy5-labeled FA-LDH (0.1 ml) via tail vein injection into a randomly selected group of mice with RA (*n* = 3). Subsequently, we captured in vivo near-infrared images of the mice through an IVIS system (BLT Aniview 100, Biolight Biotechnology, China) at pre-established time intervals (2, 4, 8, 12, 24, and 48 h).

### Histological staining

The specimens underwent a decalcification process with 10% EDTA (pH 7.4) for a duration of 4 weeks, after which they were embedded within paraffin for preservation and further analysis. Histological sections were prepared and stained with hematoxylin and eosin (H&E), toluidine blue (T&B), and safranin O-fast green (SO-FG). The stained tissue sections were scrutinized and imaged utilizing a microscope of superior quality. Synovial inflammation score was graded from 0 to 4 according to the inflammatory cell infiltration in H&E staining. To evaluate the protein expression levels associated with macrophage polarization, immunohistochemical (IHC) staining was conducted on ankle joints. The IHC protocol consisted of several steps: initial deparaffinization and rehydration of the tissue sections, antigen retrieval to unmask epitopes, and peroxidase inactivation. Following these preparatory steps, the sections were incubated with primary antibodies (IL-1β, TNF-α, and IL-10) and a secondary antibody. In our endeavor to assess the biosafety of LDH, we also processed tissues from the heart, liver, spleen, lung, and kidney of the mice. These tissues were embedded in paraffin, sectioned, and subsequently stained with H&E for further examination.

### Bone assessment and micro-CT analysis

High-resolution imaging was conducted using micro-computed tomography (CT) (Bruker Skyscan1276, USA) scanning technology. The resolution of the scanning was 10 μm; the x-ray energy was set at 50 kV and 500 μA; and fixed exposure time was 300 ms.

### Safety evaluation in vivo

To assess the safety of each formulation, serum samples were obtained from treated mice and analyzed for blood urea nitrogen (BUN) and creatinine (Cre) levels using standard assay kits, following the manufacturer’s instructions

### Statistical analysis

Quantitative data were expressed as mean ± SD from 3 independent measurements. The Student’s *t* test was employed to evaluate differences between 2 distinct groups, while one-way analysis of variance (ANOVA) was utilized to evaluate differences among multiple groups. Significance was determined at the following levels: *P < 0.05, **P < 0.01, and ***P < 0.001.

## Results

### Characterization of LDH and FA-LDH

**Fig. 2.**
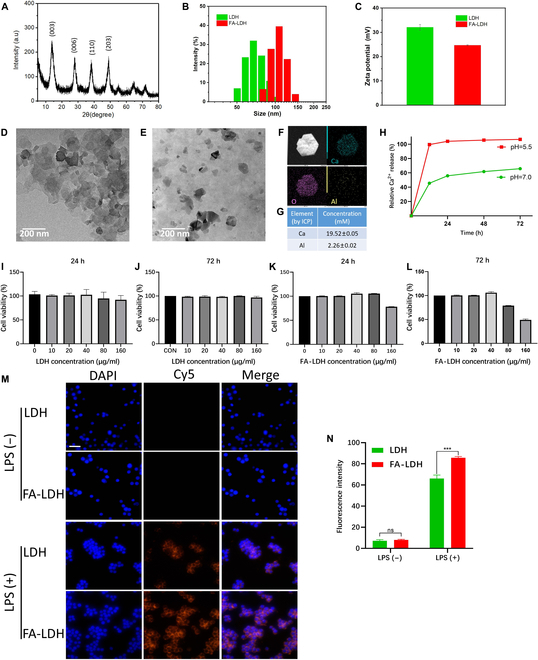
(A) XRD pattern of LDH. (B) Hydrodynamic diameter of LDH and FA-LDH. (C) Zeta potential of LDH and FA-LDH. TEM image of LDH (D and F) and FA-LDH (E). (G) ICP-OES results of LDH. (H) pH-responsive LDH degradation quantification in Ca^2+^ release percentage. (I to L) Cell viability of RAW 264.7 macrophages treated with various concentrations of LDH and FA-LDH for 24 and 72 h, respectively. (M) Confocal fluorescence imaging of macrophages treated with Cy5-labeled LDH and FA-LDH, Scale bars, 100 μm. (N) Quantitative analysis of fluorescence intensity. *n* = 3; mean ± SD; ns, not significant; ***P < 0.001.

The structure of LDH was characterized by XRD. The XRD pattern indicated a good lattice structure of LDH (Fig. 2A). The diffraction reflections (003), (006), (110), and (203) of the LDH structure can be observed in the XRD patterns [18]. The red laser was shone onto the LDH and FA-LDH aqueous dispersions sited for 1 h. A clear Tyndall effect was observed in both the LDH and FA-LDH samples, indicating the great stability of the materials (Fig. S1A). DLS measurements showed that the average hydrodynamic diameter of LDH was 69.7 ± 3.3 nm (Fig. 2B). After being modified with FA, the average hydrodynamic diameter of FA-LDH was increased to 102.1 ± 3.7 nm. Due to the negatively charged FA adsorbing onto the LDH, the zeta potential of LDH decreased from 32.07 to 24.7 mV in Fig. 2C. Phenol red is a kind of pH indicator, and its absorption intensity at 560 nm improved with the increase of pH value of solutions [18]. The effect of FA on the pH of LDH was verified by the change of color and absorption intensity at 560 nm of phenol red solution. As shown in Fig. S1B, after adding LDH and FA-LDH, the color of the phenol red solution appear to be red, like that of PBS 7.4. FA is a weak acid, and the absorption intensity at 560 nm of FA-LDH is a little lower than that of LDH. It means that the pH of FA-LDH is lower than that of LDH. TEM was performed to characterize the morphology of LDH and FA-LDH. Both LDH and FA-LDH showed an irregular hexagonal sheet structure (Fig. 2D and E). ICP-OES analysis revealed the elemental concentrations of LDH (Fig. 2G). The concentrations of Ca and Al in the LDH colloid were 19.52 ± 0.05 mM and 2.26 ± 0.02 mM, respectively, which were also confirmed by the corresponding elemental mappings in Fig. 2F. It is reported that Al^3+^ had no evident effect on the polarization of macrophages, and the biosafety of Al^3+^ remains to be verified [18]. This material has a high concentration of Ca^2+^ and a low concentration of Al^3+^, and theoretically has good biosafety. The degradation of LDH releases Ca^2+^, Al^3+^, and OH^−^. In this study, the degradation of LDH was evaluated by monitoring the release of Ca^2+^. As shown in Fig. 2H, LDH exhibited a similar degradation pattern characterized by an initial rapid phase followed by a slower phase in both acidic (pH 5.5) and neutral (pH 7.0) environments. Notably, the degradation rate of Ca^2+^ in an acidic environment (pH 5.5) was significantly higher than that in a neutral environment (pH 7.0). The degradation of LDH in the acidic environment was 87% greater than that in the neutral environment at 24 h. These results demonstrate that LDH exhibits pH-responsive degradation ability. Therefore, we hypothesize that LDH can release more OH^−^ to neutralize the acidic inflammatory microenvironment, responsively.

### Biocompatibility in vitro

The biocompatibility of LDH and FA-LDH was investigated to substantiate its potential for in vivo applications. Results from the CCK-8 assay showed that LDH exhibited nonsignificant cytotoxicity when administered at 10 to 160 μg/ml for 24 h or 72 h, compared with PBS-treated controls. The viability of RAW264.7 cells cultured in 160 μg/ml LDH were 95.5% ± 0.08% at 24 h and 96.8% ± 0.03% at 72 h (Fig. 2I and J), indicating positive biocompatibility. Calcein-AM/PI double staining was performed to show the survival status of macrophages after binding with LDH (Fig. S2). At all tested concentrations, the number of living cells was similar to that in the CON group. Cells were incubated in culture medium containing FA-LDH at the same concentrations. However, the viability of RAW264.7 cells cultured in 160 μg/ml FA-LDH was 78.23% at 24 h (Fig. 2K). A decrease in cell viability at 72 h was observed in Fig. 2L when the concentration was 80 mg/ml (78.99%) and 160 mg/ml (49.14%). Based on these data, 40 μg/ml of LDH and FA-LDH was chosen for subsequent cell experiments.

### Cellular uptake behavior

M1 macrophages demonstrate an elevated expression of folate receptors (FRs) on their surface [25]. Therefore, the surface of LDH was modified with FA to enhance the delivery of LDH to M1 macrophages by the interaction between FA and its corresponding receptor. M0 macrophages are polarized into M1 macrophages by LPS stimulation to serve as the inflammatory cell model in vitro. LDH and FA-LDH were labeled with Cy5. The internalization of LDH was observed with a fluorescence microscope (Fig. 2M). There was notable presence of red fluorescence around the cell nucleus of LPS-stimulated macrophages. Quantitative analysis of fluorescence intensity is shown in Fig. 2N. The Cy5 signal intensity in LPS-stimulated RAW 264.7 cells incubated with LDH and FA-LDH was both stronger than that in normal RAW 264.7 cells, which can be attributed to the Extravasation through Leaky Vasculature and subsequent Inflammatory cell-mediated Sequestration (ELVIS) effect of nanomaterials under inflammatory condition. Comparison of the internalization of LDH and FA-LDH in LPS-stimulated macrophages showed that the cytoplasmic fluorescence intensity of the FA-LDH group increased by approximately 30% compared with that of the LDH group. The result confirmed that FA-LDH can actively target LPS-stimulated macrophages in vitro through the interaction between FA and FR on the surface of M1 macrophages.

### Phenotypic transition of macrophages in vitro

**Fig. 3.**
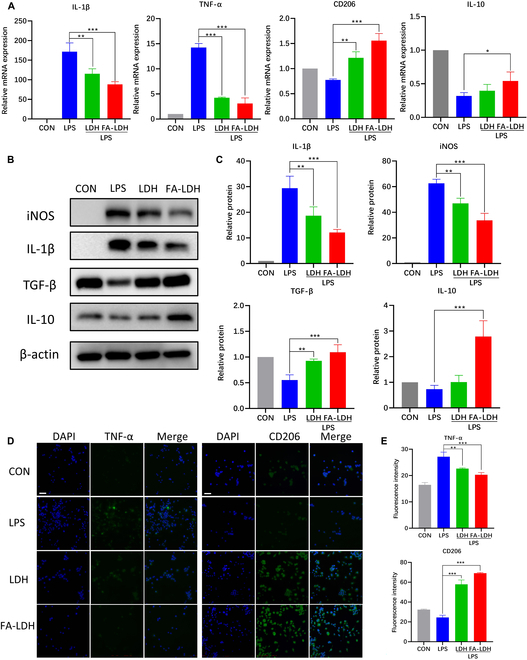
In vitro M1 to M2 polarization of macrophages by LDH and FA-LDH. (A) mRNA expression of M1 (IL-1β and TNF-α) and M2 (CD206 and IL-10) macrophage markers in RAW 264.7 cells under various conditions, as evaluated by qRT-PCR analysis. (B) Protein expression of M1 (iNOS and IL-1β) and M2 (IL-10 and TGF-β) macrophage markers in RAW 264.7 cells under various conditions, as evaluated by Western blot analysis (C). (D) TNF-α and CD206 (green), and DAPI (blue) of RAW 264.7 in different groups. (E) Quantitative analysis of the fluorescence intensity of TNF-α and CD206. Scale bars, 100 μm; *n* = 3; mean ± SD; *P < 0.05, **P < 0.01, ***P < 0.001; compared with LPS group.

Macrophage phenotypes are intimately associated with the inflammatory response in the synovium of RA [1]. Proinflammatory factors secreted by M1 macrophages exacerbate RA progression. LPS-induced macrophages were mainly attributed to M1 macrophages and used to evaluate the regulatory capacity of macrophage polarization by LDH [7]. Unstimulated macrophages were set as the control group. qRT-PCR was used to detect the mRNA expression levels of key proinflammatory markers secreted by M1 macrophages, such as IL-1β and TNF-α, and key markers of M2 macrophages (CD206 and IL-10). The results showed a marked elevation in the expression levels of IL-1β and TNF-α in the LPS group. After treatment with FA-LDH, the mRNA expression levels of IL-1β and TNF-α decreased to 52% and 24%, respectively, compared with the LPS group (Fig. 3A). Conversely, a significant suppression was observed in the levels of CD206 and IL-10 when treated with FA-LDH or LDH, and FA-LDH showed better efficiency in Fig. 3A.

Western blotting was used to corroborate the levels of protein expression (Fig. 3B and C). After LPS stimulation, the expression of iNOS and IL-1β increased significantly. Compared with the LPS group, both LDH and FA-LDH could reduce the expression of iNOS and IL-1β. FA-LDH could inhibit the expression of both inflammatory proteins with a rate of approximately 50% compared with the LPS group. Concurrently, LDH and FA-LDH increased the expression of IL-10 and TGF-β. The expression levels of IL-10 and TGF-β in the FA-LDH group increased up to ~4- and 2-fold, respectively, compared with the LPS group. Immunofluorescence staining results exhibited a similar trend (Fig. 3D and E). Compared with the LPS group, both LDH and FA-LDH reduced the fluorescence intensity of TNF-α and enhanced the fluorescence intensity of CD206. In summary, both LDH and FA-LDH possessed the ability to induce the transformation of M1 macrophages to the M2 phenotype. FA-LDH was more efficient to reprogram the macrophages, probably due to the higher internalization efficiency to M1 macrophages.

### RNA-seq verification of immunomodulation of LDH

**Fig. 4.**
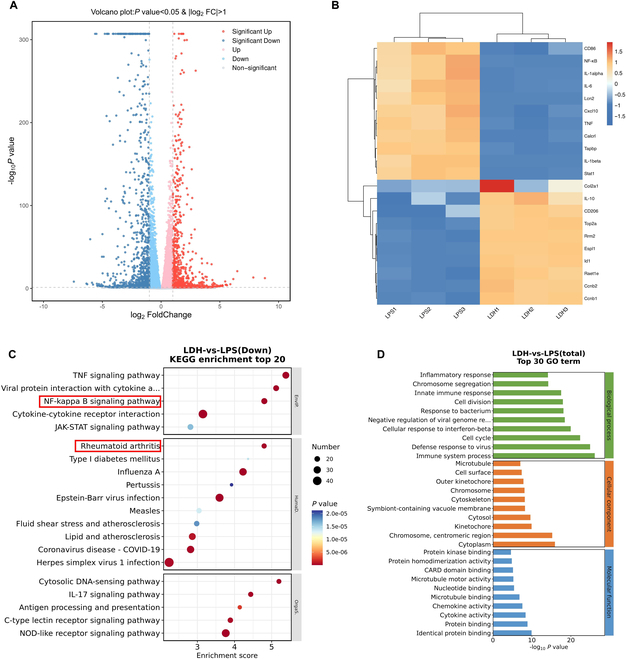
Gene expression profile of LPS-stimulated macrophages treated with or without LDH. (A) Volcano plot showing the differentially expressed genes between cells treated with or without LDH. Significant genes are colored red (up-regulated) and blue (down-regulated). (B) Expression changes of the singled-out chemokines and cytokines between LPS and LDH groups. The threshold was set as a *P* value ≤ 0.05 (*n* = 3). (C) KEGG pathway analysis between the LPS and LDH groups. (D) Gene Ontology (GO) enrichment analysis of biological process, cellular component, and molecular function.

To further explore the immunoregulatory role of LDH, RNA sequencing (RNA-seq) was performed in LPS-stimulated macrophages with or without LDH treatment. Principal component analysis was conducted on the RNA-seq data of 6 samples. This indicated that 3 independent samples revealed consistent gene expression profiles, and significant differences in gene expression were observed between 2 distinct groups (Fig. S3). A volcano plot of differentially expressed genes (DEGs) between LPS and LDH treatments showed a clear separation of macrophage gene expression, indicating significant transcriptomic reprogramming by LDH. Compared with the LPS group, 1,711 genes exhibited differential expression in the LDH group. The distribution of these DEGs in the LDH group revealed that 758 DEGs were up-regulated, whereas 953 DEGs were down-regulated, compared with the LPS group (Fig. 4A). The variations in chemokine and cytokine expression between the LPS and LDH groups were specifically isolated and regraphed (Fig. 4B). The hierarchical clustering of DEGs revealed a notable down-regulation of M1 macrophage-associated genes (CD86, IL-1β, IL-6, and TNF) within the LDH group. Conversely, genes associated with M2 macrophages, such as IL-10 and CD206, were up-regulated in the LDH group. These findings are consistent with transitions in the macrophage phenotype as confirmed above. Kyoto Encyclopedia of Genes and Genomes (KEGG) pathway analysis showed that among the deregulated pathways, the top 5 down-regulated pathways included the TNF signaling pathway, viral protein interaction with cytokine and cytokine receptor, NF-κB signaling pathway, cytokine–cytokine receptor interaction, and JAK-STAT signaling pathway (Fig. 4C). KEGG enrichment analysis revealed that down-regulated DEGs are significantly enriched in the RA pathway, indicating that LDH had the potential to alleviate RA. Gene Ontology (GO) analysis revealed in Fig. 4D that in terms of biological processes, LDH was primarily associated with immune system processes. As for cellular components, DEGs were predominantly enriched in the cytoplasm and chromosomes. In the molecular function category, DEGs were mainly involved in protein binding, cytokine activity, and chemokine activity.

### The molecular mechanism of down-regulation of the inflammation by LDH

After being stimulated by LPS, macrophages increase oxygen consumption and release a large number of protons, leading to glycolytic metabolism and lactic acid accumulation [7,26]. The pH decrease in the local microenvironment is characteristic of many chronic inflammatory diseases, such as atherosclerosis and RA [27,28]. The acid-neutralizing strategy is currently a subject of extensive research [29]. Literature indicated that both alkaline magnesium hydroxide nanoparticles and calcium-aluminum-layered double hydroxide nanosheets possess anti-inflammatory properties [17,18]. However, the underlying mechanisms remain to be elucidated. The mechanism by which macrophages sense changes in environmental pH and further modulate inflammatory pathways in a responsive manner remains unclear.

#### LDH affects the nuclear translocation of Smad5 by regulating the pH of the microenvironment

**Fig. 5.**
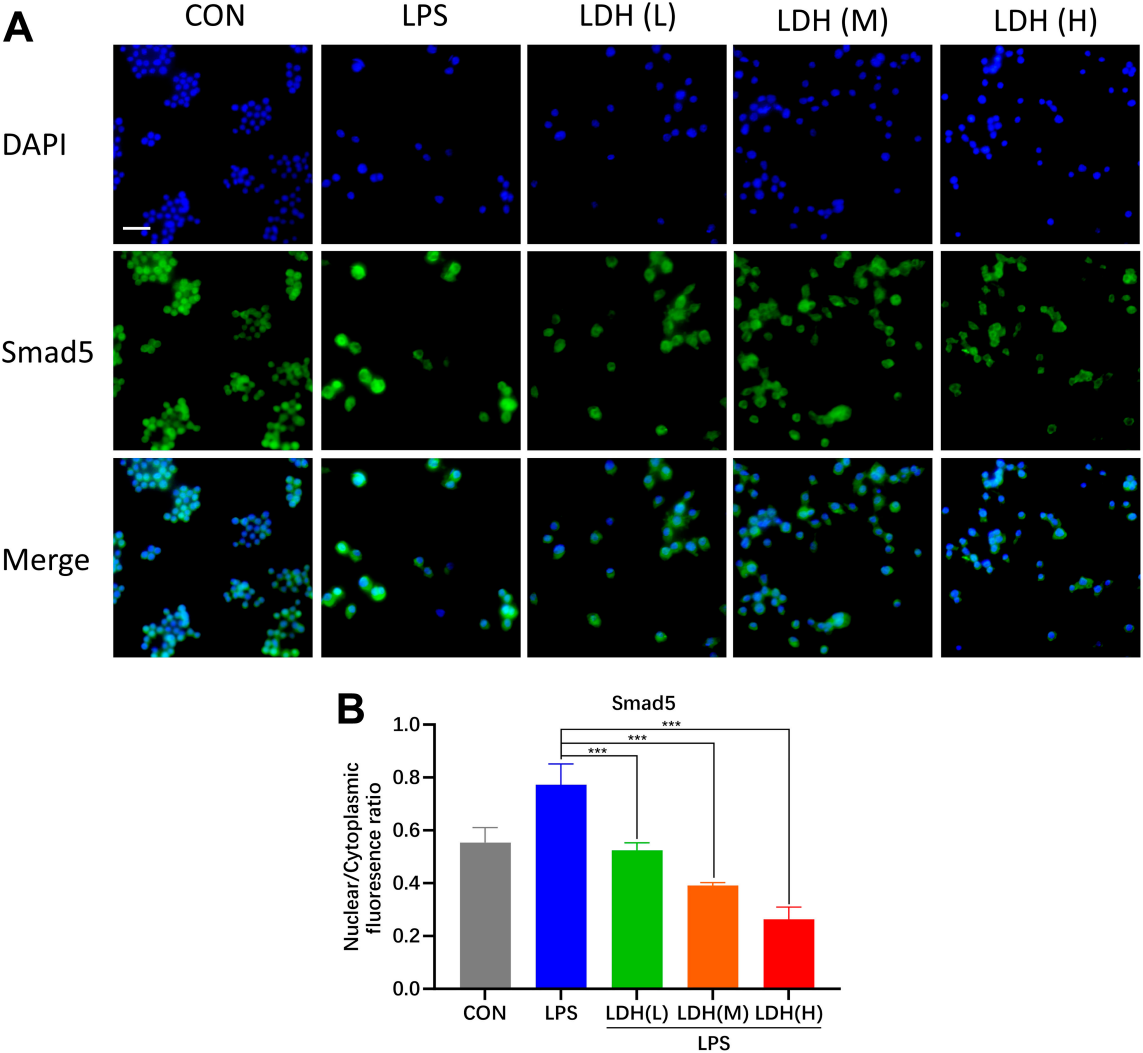
Distribution of Smad5 effected by LDH. (A) Smad5 (green) and DAPI (blue) of RAW 264.7 cultured with different groups. (B) Statistical result of nuclear and cytoplasmic fluorescence ratio of Smad5. Scale bar, 100 μm, *n* = 3; mean ± SD, ***P < 0.001.

It is reported that Smad5 was a direct intracellular pH fluctuation messenger in human pluripotent stem cells [21]. Smad5 can perceive changes in intracellular and extracellular pH and respond to the pH changes through nucleocytoplasmic shuttling. In this research, we investigated the distribution of Smad5 in LPS-stimulated macrophages. Surprisingly, the inhibition of Smad5 nuclear translocation was observed in macrophages cocultured with LDH. The degradation of LDH can functionally release metallic ion and OH^−^. Consequently, we independently investigate the influence of the pH and the variety of metal ions of LDH on the intracellular dispersion of Smad5. LDH is synthesized using CaCl_2_ or SrCl_2_ as sources of divalent metal ions, respectively. Under the same OH^−^ content, both LDH including Ca^2+^ and Sr^2+^ inhibit the nuclear translocation of Smad5, while there is no significant statistical difference between the 2 kinds of LDH (Fig. S4). It is suggested that the species of metal ions may not be the core factor influencing the intracellular distribution of Smad5. LDH with varying OH^−^ content was synthesized through modulating the addition of NaOH. LDH influenced the pH of cell culture medium in an OH^−^ concentration-dependent manner. According to different pH levels, the variants were designated as LDH(L), LDH(M), and LDH(H). Further, LDH inhibits the nuclear translocation of Smad5 with the tendency of being pH-dependent (Fig. 5A). As shown in Fig. 5B, the LDH with high OH^−^ content has the strongest inhibitory effect to the nuclear translocation of Smad5. Chloroquine (CQ) is an organic base also tested to study the effect on the distribution of Smad5. As shown in Fig. S4, CQ inhibited the nuclear translocation of Smad5 in cells. However, its inhibitory efficacy was less pronounced compared to LDH, which could be related to other biological activities of CQ. Furthermore, all kinds of LDH cannot affect the total protein expression level of Smad5 in Fig. S5. Therefore, we determined that LDH may regulate the nuclear translocation of Smad5 by modulating the pH of the microenvironment.

**Fig. 6.**
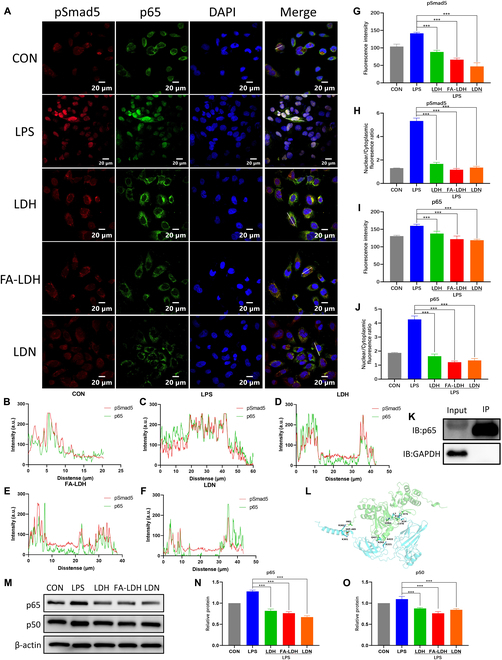
Effect of LDH and FA-LDH on the distribution and expression of pSmad5 and p65. (A) pSmad5 (red), p65 (green), and DAPI (blue) of RAW 264.7 cultured with different groups. (B to F) Fluorescence intensity profile of a set of pixels distributed on the white lines in the left merged image. (G and I) Quantitative analysis of the fluorescence intensity of pSmad5 and p65. (H and J) Statistical result of nuclear and cytoplasmic fluorescence ratio of pSmad5 and p65. (K) The interaction between pSmad5 and p65 in IP. (L) Visual analysis of the conformation for the p65 and pSmad5 complex system. (M) Western blot bands of p65 and p50. (N) Statistical result of expression levels of p65. (O) Statistical result of expression levels of p50. *n* = 3; mean ± SD; ***P < 0.001.

pSmad5 is a critical component in the activation of the Smad signaling pathway [24,30]. Upon activation of the Smad5 pathway, pSmad5 translocated to the cell nucleus to exert its function [31]. Consequently, we further investigated the impact of LDH and FA-LDH on pSmad5 (Fig. 6A). As demonstrated in Fig. 6H, the nucleocytoplasmic ratio of pSmad5 in macrophages treated with FA-LDH was reduced to 21.9% of that in the LPS group. LDN193189 (LDN) is a bone morphogenetic protein (BMP) pathway inhibitor that can suppress the phosphorylation of Smad5. In this research, LDN was introduced to investigate the biological function of Smad5 in macrophage repolarization. As shown in Fig. 6G, the fluorescence intensity of pSmad5 in the LDN group decreased to 33.1% compared to the LPS group. This indicated that LDN effectively inhibited the production of pSmad5. LDN had also been demonstrated to inhibit the nuclear translocation of Smad5 with a similar degree to LDH and FA-LDH.

#### LDH inhibits the NF-κB pathway mediated by the nuclear translocation of Smad5

We further investigated the correlation between pSmad5 nuclear translocation and the progression of cellular inflammation. It had been reported that the activation of the NF-κB pathway was directly related to nuclear translocation and phosphorylation of Smad5 [24,31]. p65 is a key molecule in the NF-κB signaling pathway. The function of the NF-κB signaling pathway largely depends on the nuclear translocation of p65 [32]. Under the stimulation of LPS, p65 translocates to the cell nucleus and promotes the transcription of target genes [33]. Zhang et al. [24] found that the overexpression of pSmad5 can activate p65. It also had been revealed that pSmad5 promoted p65 entry into the cell nucleus. Inhibiting the expression of pSmad5 can reduce the expression of p65 [31]. As shown in Fig. 6J, the nuclear–cytoplasmic ratios of p65 in the FA-LDH group were reduced to 28.3% of that in the LPS group, showing a similar trend of pSmad5. As shown in Fig. 6A, H, and J, the nuclear–cytoplasmic ratios of pSmad5 and p65 in the LDH and FA-LDH group showed a similar downward trend compared to the LPS group, and the fluorescence images of pSmad5 and p65 demonstrate a high degree of colocalization (Fig. 6B to F). The fluorescence intensity of pSmad5 and p65 was quantitatively measured. After treatment with FA-LDH, the fluorescence intensities of pSmad5 and p65 were lower than that in the LPS group in Fig. 6G and I. The interaction between pSmad5 and p65 was verified through IP in the LPS-stimulated macrophage model (Fig. 6K). Both p65 and GAPDH were present in the input group. The results showed that p65 was precipitated by pSmad5, while GAPDH did not exist in the IP group. Next, the potential binding sites between pSmad5 and p65 was explored by molecular docking techniques. The binding patterns of the 2 proteins (pSmad5 and p65) were investigated by the GRAMM protein–protein docking method. The binding free energy of the system is −9.8 kcal/mol. The butt conformation is visually shown in Fig. 6L. The interaction between pSmad5 and p65 was primarily characterized by 7 pairs of hydrogen bonds. The specific docking structures are shown in Table S2. Among them, the oxygen atom in the phosphorylated site SEP465 forms a pair of hydrogen bonds with the nitrogen atom in Lys301, suggesting that the interaction between Smad5 and p65 was further enhanced after phosphorylation of Smad5. The above results verified the interaction between p65 and pSmad5. The stimulation of LPS has been proven to activate the NF-κB signaling pathway [34]. The phosphorylation process in the NF-κB signaling pathway leads to the degradation of IκB [33]. This facilitates the translocation of p65 to the nucleus and induces the expression of target genes. In the absence of continuous activation signals, IκB exerts a negative feedback mechanism that inhibits p65 and p50 [35]. The protein expression of p65 and p50 was further analyzed by Western blot. As shown in Fig. 6N, the protein expression of p65 treated with LDH and FA-LDH was 63.9% and 59.6% of that in the LPS group. As shown in Fig. 6O, the protein expression of p50 also decreased after treatment with LDH and FA-LDH. As a key protein on the NF-κB signaling pathway, the reduction in p65 and p50 expression levels implied the suppression of the NF-κB signaling pathway. The LDN group showed similar abilities. Therefore, we postulated that LDH and FA-LDH could suppress the activation of the NF-κB signaling pathway by inhibiting the nuclear translocation of pSmad5, which restricted the nuclear translocation of p65. Given that LDN is a precise inhibitor of the BMP pathway, LDN was utilized to verify the feasibility of intervening in the Smad5 signaling pathway to regulate p65. Both FA-LDH and LDN effectively inhibited the nuclear translocation and expression of p65 and pSmad5. It has been demonstrated that the NF-κB signaling pathway is closely associated with M1 polarization of macrophages and the production of proinflammatory cytokines such as TNF-α and IL-1β [36]. Inhibiting the activation of the NF-κB signaling pathway in macrophages aids in reducing the production of proinflammatory factors and the resolution of inflammation [37]. These findings were in alignment with the aforementioned macrophages phenotypic and RNA-seq results. LDH and FA-LDH inhibited the progression of M1 macrophage-mediated inflammation by inhibiting the pSmad5/p65 axis. Subsequently, our research delves into the joint-protective capabilities of LDH and FA-LDH in vivo.

### RA-site targeting biodistribution of LDH in the CIA mouse model

**Fig. 7.**
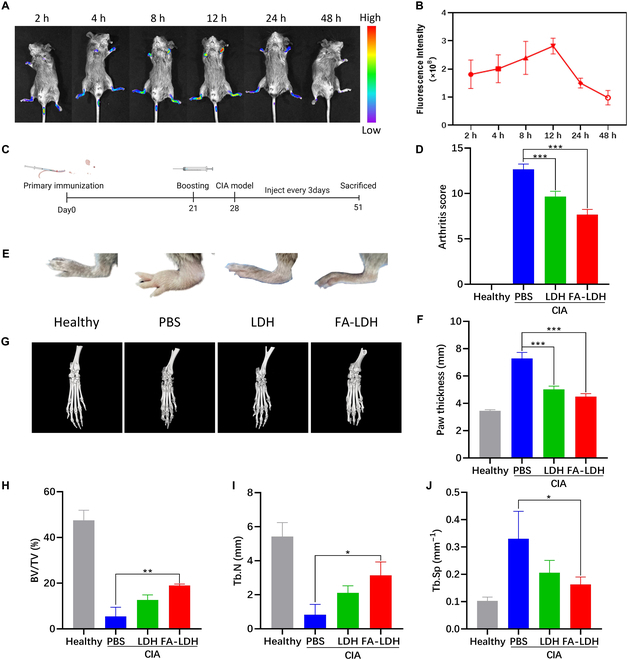
The biodistribution and therapeutic efficacy of LDH and FA-LDH in CIA mice. (A) In vivo fluorescence images and (B) statistic fluorescence intensity of FA-LDH. (C) Experimental schedule of CIA induction and treatment. (D) The arthritis score after treating for 3 weeks. (E) Representative images of hind paw. (F) The paw thickness after treating for 3 weeks. (G) Micro-CT images of hind paw. Quantitative analysis of (H) BV/TV, (I) Tb.N, and (J) Tb.Sp in each treatment group. *n* = 3; mean ± SD; *P < 0.05, **P < 0.01, ***P < 0.001.

M1 macrophages are crucial treatment targets for alleviating the symptoms of RA [15]. Therefore, we modified FA on the surface of LDH to target M1 macrophages. FR expression is up-regulated in M1 macrophages [38]. A higher abundance of FR is observed in the synovial macrophages of RA patients compared with those of healthy individuals [39]. Thus, it might lead to an enhanced delivery capability of FA-LDH to inflamed joints. To assess the targeting efficiency of FA-LDH in CIA mice, Cy5-FA-LDH was injected through the caudal vein, and fluorescence was detected using IVIS (Fig. 7A). The accumulation of fluorescence intensity is recorded in Fig. 7B. Fluorescence predominantly accumulated in distal small joints, such as the ankle or metacarpophalangeal joint, as early as 2 h after administration, indicating an obvious inflammatory joint-targeting capability of FA-LDH. Fluorescence intensity at the distal joints of limbs progressively increased and remained strongest at 12 h in the inflamed paws (Fig. 7B). After 48 hours, almost no specific fluorescence signal was observed in the limbs of the mice. These results indicate that FA-LDH had an effective accumulation performance in the inflamed joints. This finding underscores the potential of FA-LDH as a targeted therapeutic agent for RA.

### Therapeutic effect on the CIA mouse model

The CIA model, which mirrors both the immunological and pathological features of RA, has been widely employed as a representative model for elucidating the pathogenesis of RA and appraising potential therapeutic strategies [40]. The anti-RA potential of LDH was assessed in CIA mice. Seven days after the second immunization, the CIA mice were administered injections with 100 μl of PBS, LDH (5 ml/kg), or FA-LDH (5 ml/kg) every 3 days for 3 weeks (Fig. 7C). Healthy mice were used as controls. The severity of RA in clinical evaluation was determined by clinical arthritis score and paw thickness. The swelling of limbs shrank considerably after treatment (Fig. 7E). The value of arthritis score was 12.67 ± 0.58 in the PBS group, 9.67 ± 0.58 in the LDH group, and 7.67 ± 0.58 in the FA-LDH group (Fig. 7D). As shown in Fig. 7F, the mean paw thickness was 5.02 ± 0.25 mm within the LDH group. In the FA-LDH group, the mean paw thickness was 4.50 ± 0.22 mm. Paw thickness of CIA mice treated with LDH and FA-LDH exhibited a significant difference compared with the PBS group. Bone erosion is a critical factor that contributes to disease severity and poor prognosis. Three-dimensional bone micro-CT was utilized to ascertain the efficacy of LDH and FA-LDH in mitigating bone erosion (Fig. 7G). Micro-CT analysis revealed that the ankle joint of CIA mice exhibited a rough bone surface and severe bone erosion. Compared with the PBS group, mice treated with LDH and FA-LDH had smoother bone surfaces and higher bone density. Quantitative analysis of micro-CT data was measured from the trabecular bone of the hind paw calcaneus. In the PBS group, percent bone volume (BV/TV) was on average 5.47% (Fig. 7H), trabecular number (Tb.N) was on average 0.83 mm (Fig. 7I), and trabecular separation (Tb.Sp) was on average 0.33 mm^−1^ (Fig. 7J). Minimal bone erosion was observed in the ankle joints of mice treated with FA-LDH. These indices were significantly improved in the FA-LDH group: BV/TV was 19.00% ± 0.61%, Tb.N was on average 3.14 mm, and Tb.Sp was 0.16 ± 0.03 mm^−1^. It is indicated that FA-LDH effectively alleviated the progression of bone damage and reversed bone erosion from RA.

**Fig. 8.**
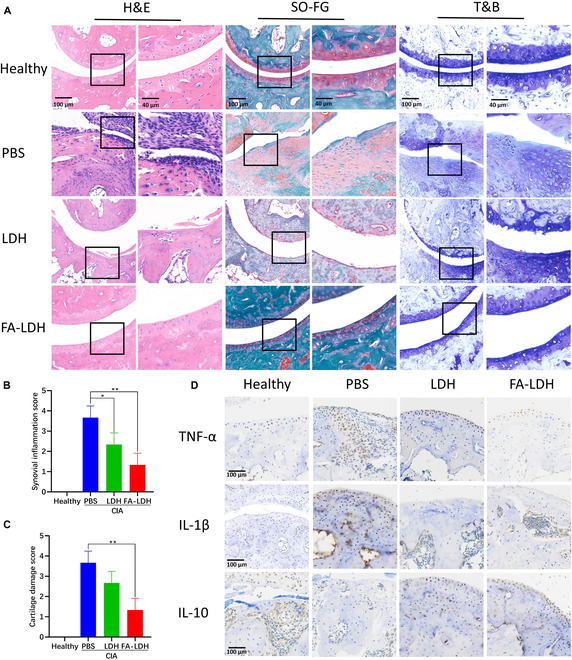
Pathological staining with (A) H&E, SO-FG, and T&B. (B) Synovial inflammation score and (C) cartilage damage score according to histopathology in joints. (D) IHC staining for visualization of TNF-α, IL-1β, and IL-10. *n* = 3; mean ± SD; *P < 0.05, **P < 0.01.

Histological analysis was performed on sectioned ankle joints of mice. In Fig. 8A, H&E staining demonstrated notable joint tissue damage and a disrupted articular cartilage surface in the PBS group, accompanied by extensive infiltration of inflammatory cells in the soft tissues surrounding the joint capsule, synovial hyperplasia, and severe bone tissue destruction. In contrast, the FA-LDH group presented with a smooth articular surface and only minor tissue damage. The soft tissues around the joint capsule exhibited minimal inflammatory cell infiltration, and the synovium showed mild hyperplasia. To ascertain the alterations in cartilage concomitant with the inflammatory cell infiltration and joint destruction, SO-FG and T&B staining was performed. The cartilage exhibits a red coloration upon interaction with the basic dye, safranin O. Cartilage cells stained with T&B exhibit a purple-blue coloration. In the healthy group, the cartilage remained intact. In contrast, CIA mice displayed thinning and erosion of joint cartilage tissue. Compared with the PBS group, LDH and FA-LDH groups demonstrated an increase in the area of cartilage tissue stained with safranin O and T&B. This suggests the capacity of LDH in mitigating synovitis and cartilage degradation in CIA mice. The FA-LDH group has a better therapeutic effect than the LDH group probably due to the targeting performance derived from FA. The severity of RA was also assessed using the synovial inflammation score. The synovial inflammation score of the FA-LDH group decreased ~60% compared with the LPS group (Fig. 8B). Additionally, the score of cartilage damage was 3.67 ± 0.58 in the PBS group and 1.33 ± 0.58 in the FA-LDH group (Fig. 8C). It is also revealed that FA-LDH had a superior effect on anti-inflammatory as well as bone and cartilage protection among all groups. IHC staining shown in Fig. 8D aims to evaluate the presence of proteins related to macrophage polarization in ankle joints. In the PBS group, the intensity and distribution of TNF-α and IL-1β staining were notably increased. This indicates that CIA mice lead to M1 macrophage polarization. In contrast, in treatment groups, particularly in the FA-LDH group, the positive staining for TNF-α and IL-1β was markedly reduced. This suggests that LDH and FA-LDH can effectively inhibit M1 macrophage polarization. In the PBS group, IHC staining showed an obvious decrease in IL-10 expression. Conversely, treatment with LDH and FA-LDH resulted in an increase in the density of IL-10-positive cells. This indicates an evident role for LDH and FA-LDH in promoting M2 macrophage polarization. Collectively, these results indicate that the LDH synergizing with FA can effectively reduce joint inflammation, inhibit M1 polarization, and promote M2 polarization of macrophages in joint, which is promising for the drug-free therapy of RA.

### Safety evaluation of LDH in vivo

**Fig. 9.**
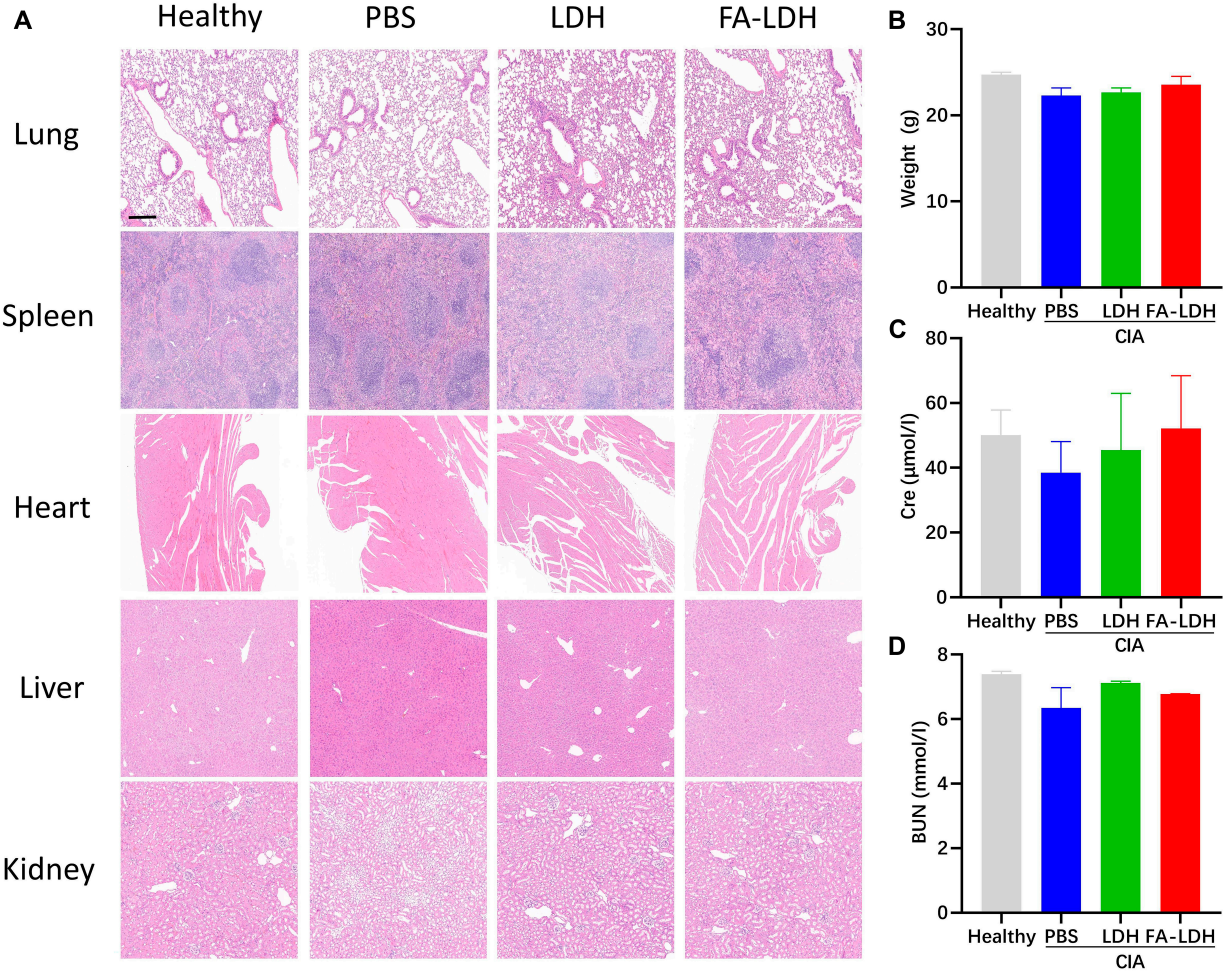
In vivo safety of LDH. (A) H&E staining of major organs in different groups; scale bar, 200 μm. (B) Body weight of the mice at day 51. (C) Cre and (D) BUN level of the mice at day 51. *n* = 3; mean ± SD.

A paramount concern associated with nanomaterial-based therapies is the potential toxicity induced in normal tissues. Throughout the experimental duration, mice in the LDH and FA-LDH groups exhibited no clinical discomfort prior to euthanasia. There were no significant alterations in body weight observed across the different groups (Fig. 9B). Major organ histology (lung, spleen, heart, liver, and kidney) and blood biochemistry examinations were conducted following various treatments to evaluate the in vivo biosafety of LDH and FA-LDH. Upon examination, no noticeable differences in histological morphology were discernible among the LDH, FA-LDH, and healthy groups (Fig. 9A). Given that the kidneys serve as the primary organs for the clearance of nanomaterial, a further evaluation of nephrotoxicity was undertaken [40]. Compared with the healthy group, neither the LDH group nor the FA-LDH group had any aberrant changes in renal function indices, including BUN (Fig. 9C) and Cre (Fig. 9D). These results suggest that LDH and FA-LDH did not exhibit obvious toxicity in major organs, underscoring their potential as safe therapeutic agents.

## Discussion

RA is a systemic autoimmune disorder, which initially manifests in the small joints of the hands and feet, before progressing to larger joints [5]. Immunosuppressants, including traditional medications, such as methotrexate, and novel biologics, such as adalimumab, are used for treating RA [3]. However, owing to the inadequate specificity of targeting and the pronounced side effects, the present therapeutic strategies for RA exhibit obvious limitations [41]. The immunomodulatory properties of bioactive materials have garnered widespread attention [42,43]. In this study, LDH was used as therapeutic intervention for RA and its immunomodulatory role was investigated. Compared with traditional immunosuppressants, LDH possesses advantages such as easy synthesis, reduced toxicity, and simple ionic composition.

Although the mechanisms of RA are elusive, insights into its pathogenesis suggest that macrophages undergo extensive infiltration and play an important role during the development and exacerbation of RA [44]. Macrophages express many cell surface receptors for the recognition of intercellular signals [45]. These receptors can be triggered by specific stimuli, leading to further polarization of macrophages into either proinflammatory M1 macrophages or anti-inflammatory M2 macrophages [7]. M1 macrophages are characterized by increased expression of iNOS, TNF-α, and IL-1β, which can further exacerbate M1 polarization of macrophages and aggravate inflammation [15]. In RA, large numbers of M1 macrophages are recruited to and infiltrate sites of inflammation [46]. The release of inflammatory factors exacerbates RA progression. M2 macrophages can release anti-inflammatory factors, like IL-10 and TGF-β [7]. Fully polarized M1 and M2 macrophages may undergo polarization reversal within several days [8]. The influence of LDH on macrophage polarization at both the protein and gene levels in this study was substantiated. The RNA-seq results verified that LDH treatment decreased the expression of macrophage-associated inflammatory genes (such as TNF, CD86, IL-1β, and IL-6). Furthermore, it was indicated that LDH reduces the expression of M1 markers (TNF-α, IL-1β, and iNOS) in macrophages by qRT-PCR and Western blot. Besides, the LDH could increase the expression of M2 markers (CD206, TGF-β, and IL-10). IL-10 can inhibit M1 macrophage polarization and reduce the production of inflammatory IL-1β and TNF-α [10]. In this context, the conversion of M1 macrophages to M2 macrophages represents a promising approach for treating inflammation. The NF-κB signaling pathway is an important target for inflammatory responses and has emerged as one of the most extensively studied transcriptional regulatory systems [37]. The NF-κB signaling pathway is up-regulated significantly within the inflammatory environment. Upon activation of the NF-κB pathway, inflammatory TNF-α and IL-1β are released, which is closely associated with the M1 polarization of macrophages and contribution to RA progression. In contrast, inhibition of NF-κB signaling induces repolarization toward M2 macrophages [15]. Note that LDH might regulate the immune microenvironment of RA by inhibiting NF-κB signaling based on the results of our experiments in vitro.

Studies have demonstrated that inflammatory microenvironments can induce a range of alterations in cellular homeostasis, like metabolic acidosis [47]. The onset of metabolic acidosis could change the pH in the immune microenvironment. Acidification of the cellular microenvironment can impact cell function and phenotype, exacerbating pathological processes [29]. LDH can degrade and release OH^−^ to neutralize the acidic microenvironment prevalent in inflammatory conditions. Next, Smad5 senses pH changes in the cellular microenvironment and initiates a response [21]. In this study, it was found that LDH can influence the intracellular distribution of Smad5 by modulating pH in the immune microenvironment. It is worth noting that co-directional interactions exist between the Smad and NF-κB signaling pathways [24,31]. This interaction was also validated through colocalization, IP, and molecular docking in this work (see the “LDH inhibits the NF-κB pathway mediated by the nuclear translocation of Smad5” section). In this way, LDH not only inhibits the nuclear translocation of Smad5 and pSmad5, but also inhibits the nuclear translocation of p65. The NF-κB signaling pathway is activated through the phosphorylation of IκB, leading to the degradation of IκB [33]. This allows for the translocation of p65 to the nucleus and the activation of inflammatory gene transcription (such as TNF-α and IL-1β) [48]. The expression of these inflammatory genes, in turn, stimulates the activation of the NF-kB signaling pathway [49]. Consequently, the nuclear translocation of p65 is a critical step in the activation of NF-κB signaling. LDH can inhibit the nuclear translocation of p65 and, thus, block the activation of NF-κB signaling. In the absence of persistent activation signals, IκB serves as negative feedback that sequesters p65 to terminate signal transduction [33]. In subsequent experiments, significant down-regulations of p65 and pSmad5 were observed following LDH treatment in LPS-stimulated macrophages. It was verified that LDH could inhibit the NF-κB signaling pathway. It is worth noting that the phosphorylation of Smad5 was not influenced by pH in normal human pluripotent stem cells [21]. However, in this research, evident inhibition of Smad5 phosphorylation was observed, showing a different regulatory mechanism. This could probably be attributed to the diversity of cell types and the unique characteristics of the immune microenvironment. An LPS-stimulated inflammatory macrophage model was used in this study, and the NF-κB and BMP signaling pathways were activated in this inflammatory model. LDH inhibits the nuclear translocation of p65, thereby suppressing the activation of the NF-κB signaling pathway, whose feedback could inhibit BMP signaling through the interaction between pSmad5 and p65, resulting in the suppression of Smad5 phosphorylation. To further explore the role of pSmad5 in this mechanism, LDN was also introduced in the LPS-stimulated inflammatory macrophage model. LDN, an inhibitor of the BMP pathway, can suppress Smad5 phosphorylation, thereby inhibiting the nuclear translocation of Smad5. The results indicated that LDN exhibited a similar biological function and anti-inflammatory effects to LDH. Both LDN and LDH can suppress the nuclear translocation and phosphorylated expression of Smad5 and p65. LDH, as an inorganic biomaterial, demonstrates superior biosafety and lower usage costs compared with the cytokine inhibitor, providing a promising candidate for clinical development.

FR is overexpressed on the membrane of M1 macrophages [25,38]. To further enhance the targeted delivery of LDH, FA was modified on the surface of LDH. The interaction between FA and FR would promote the cellular uptake of FA-LDH. In vitro experimental results confirmed that the FR-mediated uptake pathway plays a vital role in delivering FA-LDH into M1 macrophages, which is of remarkable importance for better targeted delivery of LDH to RA joints. The in vivo results showed that FA-LDH had good targeting and retention ability toward inflammatory joints. Further, FA-LDH exhibited superior therapeutic efficacy in the CIA mouse model and reduced synovial tissue damage with no organ toxicity.

## Conclusion

This study demonstrates the positive role of LDH in RA treatment. LDH serves as a key modulator in the inflammatory response of LPS-induced RAW 264.7 cells by inhibiting the nuclear translocation of pSmad5 and p65, therefore suppressing the activation of NF-κB signaling and inducing polarization of macrophage from M1 to M2. Consequently, the inflammatory microenvironment was reshaped by LDH. In the CIA model, LDH treatment eliminated swelling and inflammation of the affected joints, effectively protecting the joint cartilage and subchondral bone structure. In vitro and in vivo observations indicated that FA-LDH exhibits satisfactory targeting ability to activated macrophages and substantial accumulation in inflamed joints. In summary, FA-LDH shows powerful biological function and great potential in drug-free therapy of RA.

## Data Availability

All data generated or analyzed during this study are included in this article.
