# Supplementary material for: Layered Double Hydroxide Reshapes the Immune Microenvironment of Rheumatoid Arthritis through Small Mothers against Decapentaplegic 5
Source: Biomater Res. 2025 Mar 28;29:0176. doi: 10.34133/bmr.0176 (PMC11951257; doi:10.34133/bmr.0176)
Supplement: Supplementary 1 — Figs. S1 to S5 Tables S1 and S2 [file bmr.0176.f1.docx]

Layered Double Hydroxide Reshapes Immune Microenvironment of Rheumatoid Arthritis through Small Mothers Against Decapentaplegic 5

Dengju Li ^1,†^, Yawei Sun ^5,†^, Guangxian Liu ^3^, Changxing Liu ^2^, Guojiang Zhang ^2^, Haojue Wang 2, Shui Sun ^1, 2, 4, *^，Senbo An ^1, *^

1 Department of Joint Surgery, Shandong Provincial Hospital Affiliated to Shandong First Medical University, Jinan, Shandong, 250021, China

2 Department of Joint Surgery, Shandong Provincial Hospital, Shandong University, Jinan, Shandong, 250012, China

3 Department of Orthopaedic, Shandong Provincial Hospital Affiliated to Shandong First Medical University, Jinan, Shandong, 250021, China

4 Orthopaedic Research Laboratory, Medical Science and Technology Innovation Center, Shandong First Medical University & Shandong Academy of Medical Sciences, Jinan, Shandong, 250117, China

5 Shandong Key Laboratory of Reproductive Medicine, Department of Obstetrics and Gynecology, Shandong Provincial Hospital Affiliated to Shandong First Medical University, Jinan, Shandong 250021, China

**Table S1.** Primer sequences used for RT-qPCR.


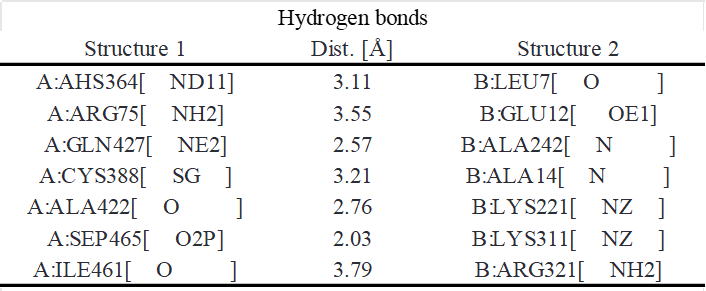


**Table S2.** Specific docking structures.

**
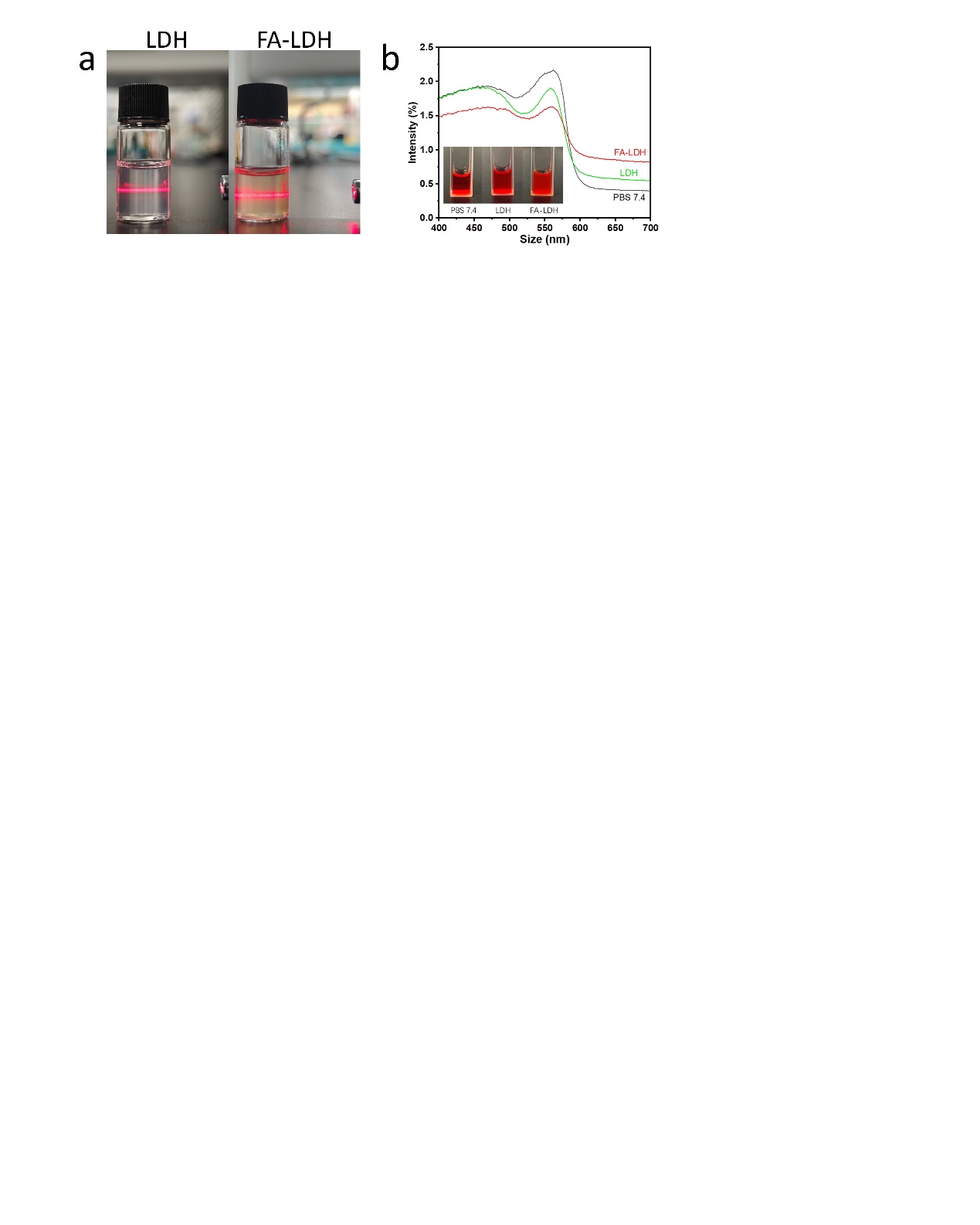
Figure S1.** (a) Tyndall effect of LDH and FA-LDH. (b) Color and UV-vis spectra changes of phenol red solution after the addition of PBS 7.4, LDH and FA-LDH.


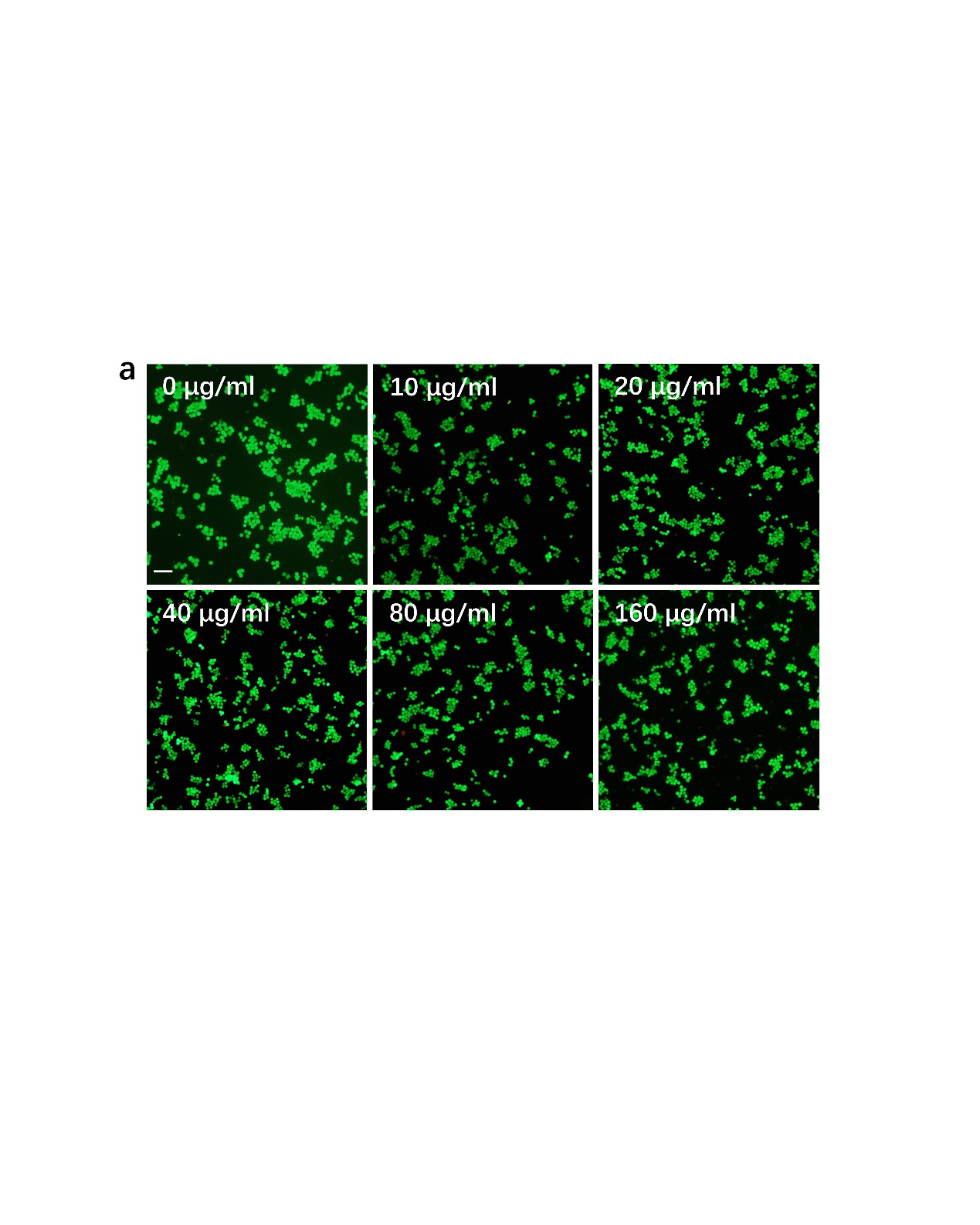
**Figure S2.** Live/dead staining of RAW 264.7 macrophages treated with various concentrations of LDH for 24 h. Scale bars, 100 μm.

**
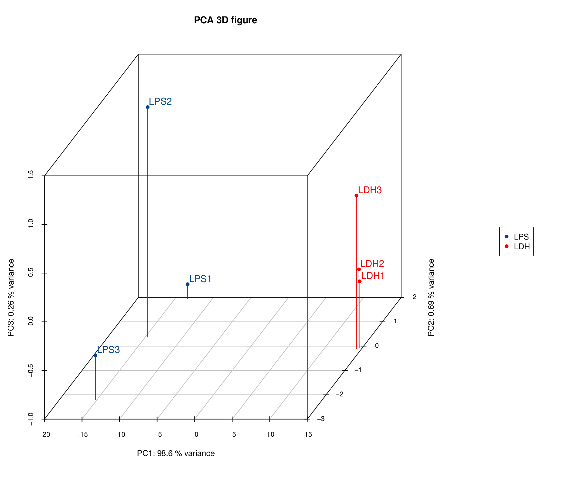
Figure S3.** Results from Principal component analysis (PCA) on all samples.

**
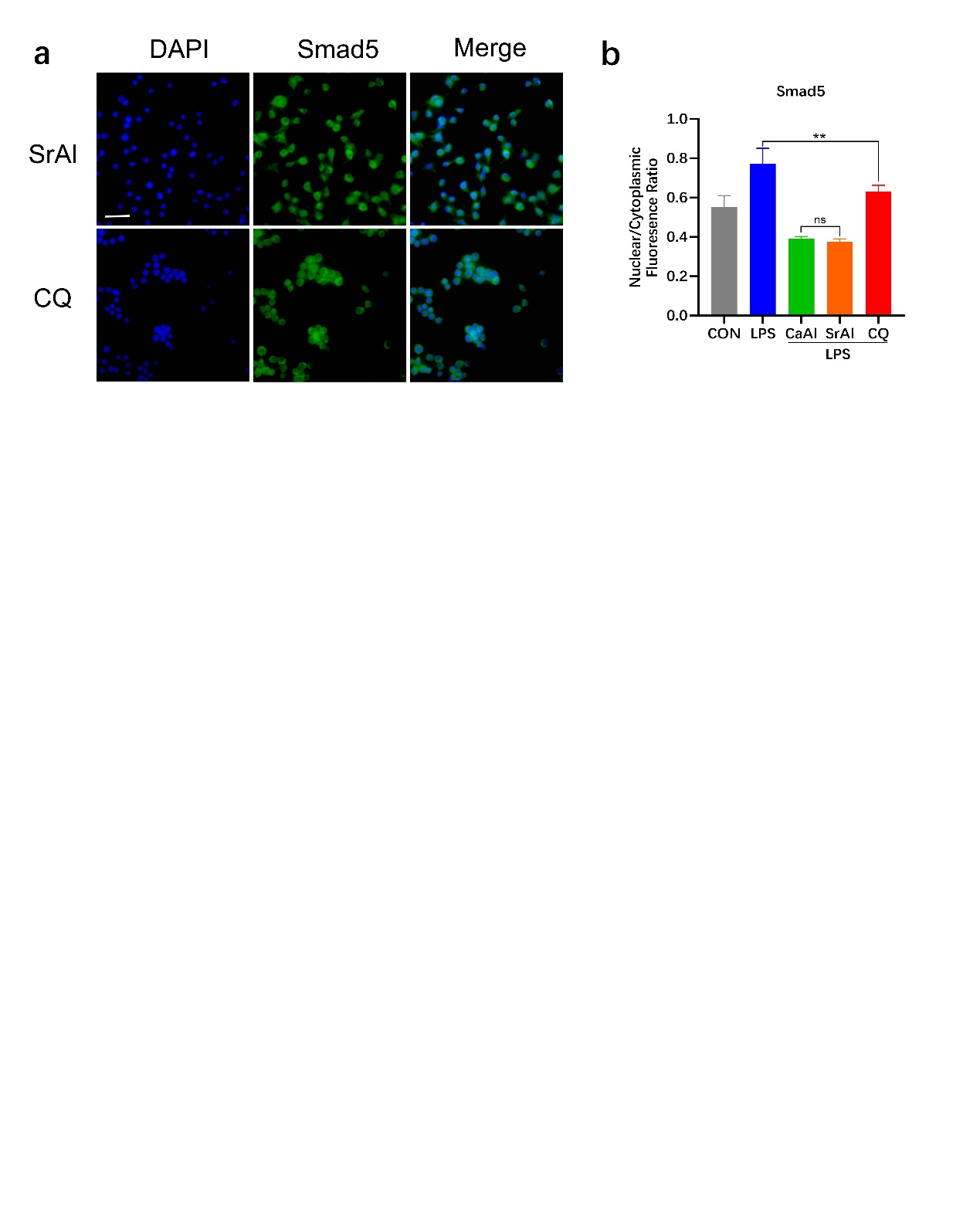
Figure S4.** The influence of metal ions and chloroquine (CQ) on the intracellular distribution of Smad5. (a) Smad5 (green), and DAPI (blue) of RAW264.7 treated with Sr^2+^ and CQ. Scale bars, 100 μm. (b) Average fluorescence quantification of nuclear and cytoplasmic Smad5 (n = 3; mean ± SD; ns, not significant; **p < 0.01).


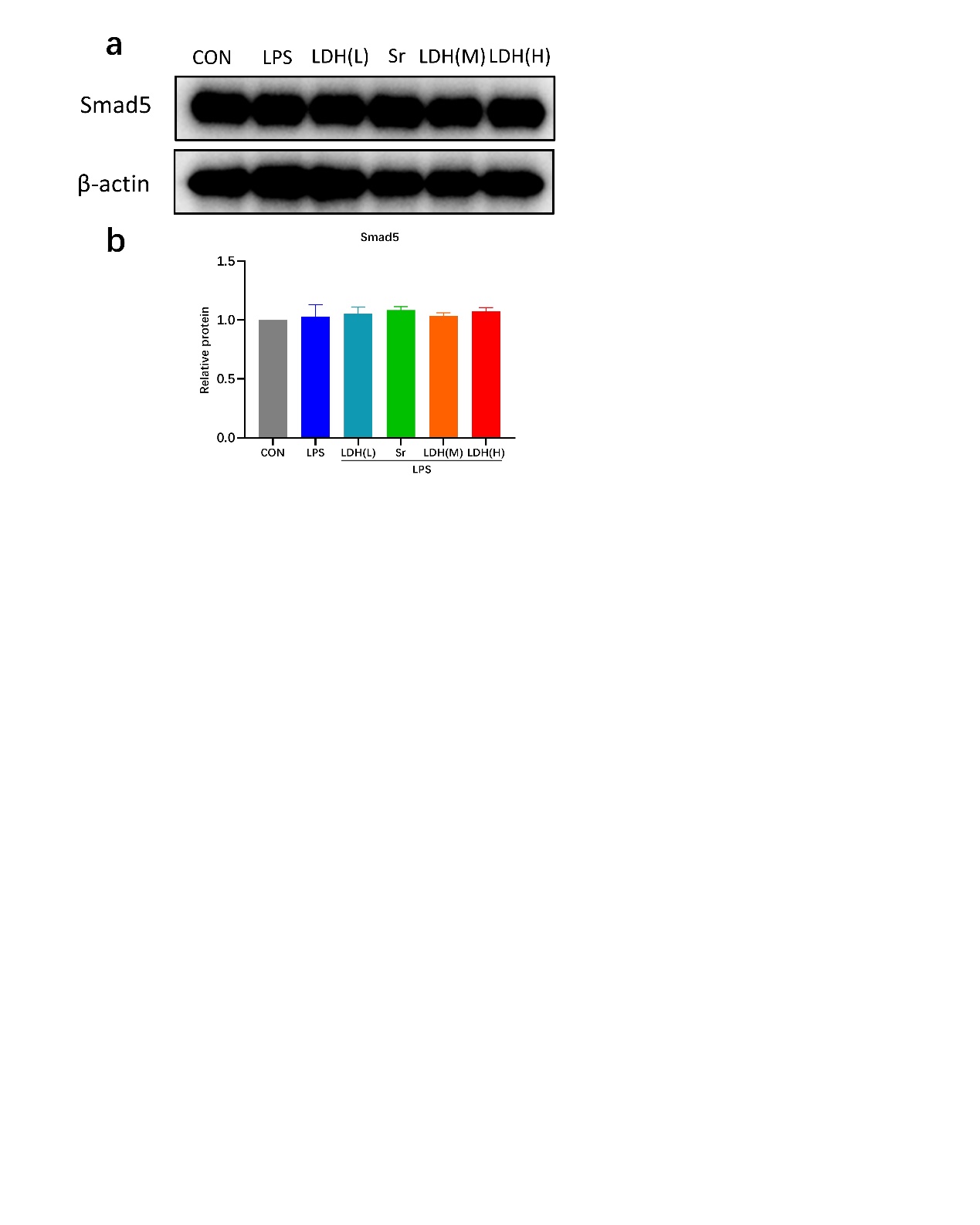
**Figure S5.** (a) Total expression level of Smad5 under various pH and metal ion conditions. (b) Statistical result of expression levels of Smad5.
